# The Regulatory Role of Pancreatic Enzymes in the Maintenance of Small Intestinal Structure and Enterocyte Turnover with Special Reference to Alpha Amylase

**DOI:** 10.3390/ijms26010249

**Published:** 2024-12-30

**Authors:** Kamil Zaworski, Piotr Wychowański, Dominika Szkopek, Jarosław Woliński, Janine Donaldson, Stefan Pierzynowski, Kateryna Pierzynowska

**Affiliations:** 1 Department of Animal Physiology, The Kielanowski Institute of Animal Physiology and Nutrition, Polish Academy of Sciences, Instytucka 3, 05-110 Jabłonna, Poland; 2 Anara AB, Alfagelgranden 24, 231 32 Trelleborg, Sweden; wychowanski@o2.pl (P.W.); janine.donaldson@wits.ac.za (J.D.); stefan.pierzynowski@biol.lu.se (S.P.); 3 Division of Oral Surgery and Implantology, Department of Head and Neck and Sensory Organs, Institute of Clinical Dentistry, Gemelli Foundation for the University Policlinic, Catholic University of the “Sacred Heart”, 00168 Rome, Italy; 4 Department of Oral Surgery, Medical University of Gdańsk, M. Skłodowskiej-Curie 3a, 80-210 Gdańsk, Poland; 5 Large Animal Models Laboratory, The Kielanowski Institute of Animal Physiology and Nutrition, Polish Academy of Sciences, Instytucka 3, 05-110 Jabłonna, Poland; d.szkopek@ifzz.pl (D.S.); j.wolinski@ifzz.pl (J.W.); 6 School of Physiology, Faculty of Health Sciences, University of the Witwatersrand, Parktown, Johannesburg 2193, South Africa; 7 Department of Biology, Lund University, 221 00 Lund, Sweden; 8 Department of Medical Biology, Institute of Rural Health, Jaczewskiego 2, 20-090 Lublin, Poland

**Keywords:** alpha amylase, pancreatic enzymes, exocrine pancreatic insufficiency, small intestine, enterocyte turnover, peptide transporter-1

## Abstract

The aim of this study was to elucidate the impact of porcine pancreatic enzymes (Creon^®^ pancrelipase) in comparison to microbial-derived alpha amylase (MD amylase) on the small intestine wall structure, mucosal glycogen accumulation, and enterocyte turnover. The impact of enzyme supplementation on the small intestine was explored in 18 pigs with surgically induced exocrine pancreatic insufficiency (EPI). Four healthy pigs served as the control group. EPI led to reduced villus length, crypt depth, and thickness of the mucosa and muscularis layers compared to those of healthy pigs. All these changes appeared to be reversible after enzyme supplementation. Brush border thickness was decreased in EPI and increased with both enzyme preparations, with MD amylase treatment leading to the highest values in the proximal jejunum. No EPI-induced changes were observed in the goblet cell (GC) population, but significant increases in GC number and area were observed following MD amylase treatment. Glycogen accumulation within the duodenal mucosa was significantly increased in EPI pigs. EPI was also shown to significantly increase apoptotic activity and decrease proliferative activity in comparison to healthy animals, while both enzyme preparations resulted in the complete recovery of both proliferative and apoptotic activity in all investigated intestinal segments. Creon^®^ influenced the morphology of the small intestine. However, supplementation of exogenous microbial amylase alone also affected gut morphology in a similar way to that of the complex host pancreatic enzymes offered orally. These data indicate that in addition to their role in digestion of nutrients in EPI, intraluminal pancreatic enzymes, especially amylase, contribute to gut health through maintenance of the intestinal wall architecture and physiological enterocyte turnover.

## 1. Introduction

The digestion, absorption, and metabolism of macronutrients, such as fats, proteins, and carbohydrates, is a complex and multi-step physiological process, in which the pancreas plays a key role [1,2]. The lack of pancreatic enzymes, or their reduced activity, of various etiologies, results in the development of exocrine pancreatic insufficiency (EPI). EPI causes poor digestion, malabsorption, and malnutrition, and ultimately leads to growth deprivation in young organisms [3,4]. Lack of pancreatic enzymes, leading to the inadequate digestion of food, results in reduced nutrient utilization, which in turn causes the accumulation of nutrient leftovers in the intestinal lumen, followed by changes in the gut microbiota [5,6,7]. In addition, pancreatic enzymes have been shown to possess extra-digestive functions affecting the intestinal mucosa [8,9,10,11]. The mixture of porcine pancreatic enzymes (Creon^®^) was shown to influence intestinal structure and accelerate gut maturation [9,12]. Recently, amylase was shown to enhance cell proliferation and differentiation and thus influence the structural and functional remodeling of the small intestine mucosa in the duodenum [13]. Moreover, pancreatic amylase actively participates in glucose metabolism and reduces both insulin release and secretion [14,15,16].

It has been shown that piglets undergoing PDL (pancreatic duct ligation) surgery to induce EPI, before 2 months of age (but not when PDL is performed at 3 months of age), display growth inhibition in the absence of pancreatic enzyme replacement therapy (PERT) [17,18,19]. Moreover, even when being fed parenterally with classical total parenteral nutrition (TPN), these piglets do not grow [4] unless pancreatic enzymes are provided enterally. The PERT, used as a treatment for EPI, aims to restore normal digestion and achieve and maintain an adequate nutritional status [20]. Based on clinical studies and extensive clinical experience, PERT is considered to be reasonably well tolerated [21,22]. This confirms that pancreatic enzymes perform regulatory functions in addition to their direct involvement in digestion.

MD amylase has been used to treat EPI [11,16,23] but still little is known about the influence and importance of particular pancreatic enzymes for the maintenance and protection of proper intestinal structure and enterocyte turnover. The possible gut-driven mechanisms underlying reduced glucose absorption after amylase supplementation also remain unclear. Hence, our study aimed to elucidate the impact of MD amylase in comparison to the mixture of porcine pancreatic enzymes (pancrelipase) on intestinal wall structure, mucosal glycogen accumulation, enterocyte turnover, and selected brush border enzymes and receptors in the small intestine in an EPI pig model.

## 2. Results

### 2.1. Structural Changes in Small Intestine

The main investigated parameters of small intestinal wall morphology, such as mucosa thickness, villus length, crypt depth, and muscularis thickness, are shown in Table 1. The mucosa thickness in the duodenum and proximal jejunum was not affected by EPI status and/or enzyme supplementation. At the same time, villus length, crypt depth, and lamina muscularis thickness were significantly decreased in EPI animals compared to the healthy group (*p* < 0.05). All of the above-mentioned parameters were restored to the values observed in healthy animals by both types of enzyme supplementation (MD amylase or Creon^®^, *p* < 0.05) (Table 1). The mucosa thickness in the middle jejunum was not affected by EPI status when compared to healthy animals, but both MD amylase and Creon^®^ supplementation increased the mucosa thickness in EPI animals. The values of mucosa thickness in the middle jejunum in both enzyme-treated groups were significantly (*p* > 0.05) higher than those observed in the healthy control group (Table 1).

In the middle jejunum, villus length tended to be lower in EPI animals (*p* < 0.1) compared to the healthy group of pigs. Both MD amylase and Creon^®^ supplementation led to a significant (*p* < 0.05) increase in villus length of EPI pigs. In Creon^®^-treated animals, villus length was even higher than that observed in the healthy pigs (by 8%, *p* < 0.05, Table 1).

There was no difference in crypt depth in the middle jejunum of healthy and EPI animals. MD amylase supplementation tended to increase this parameter, while Creon^®^ treatment led to a significant (*p* < 0.05) increase in crypt depth by 30% in EPI animals. At the same time, lamina muscularis thickness was significantly decreased in EPI animals compared to the healthy control group (by 63%, *p* < 0.05). This parameter was restored to values observed in healthy animals by both types of enzyme supplementation (MD amylase or Creon^®^, *p* < 0.05) (Table 1).

The thickness of the mucosa in the distal jejunum was not affected by EPI status and/or enzyme supplementation. In this intestinal segment, the villus length in EPI animals was not different from that in the healthy animals (Table 1), and MD amylase treatment tended to increase this parameter, while Creon^®^ supplementation led to a significant increase in villus length in EPI animals. A similar situation was observed for crypt depth, which was not affected by EPI status, but significantly increased by enzyme supplementation (both amylase and Creon^®^ treatment, Table 1). At the same time, in the distal jejunum, lamina muscularis thickness tended to be lower in EPI animals when compared to the healthy group (by 36%, *p* = 0.1). This parameter was restored to the values observed in healthy animals by both types of enzyme supplementation (MD amylase or Creon^®^, *p* < 0.05) (Table 1).

The mucosa thickness in the ileum was not affected by EPI status and/or enzyme supplementation. The villus length in EPI animals tended to be lower when compared to healthy animals (Table 1). At the same time, both crypt depth and lamina muscularis thickness were significantly (*p* < 0.05) lower in EPI animals compared to the healthy group (by 24% and 44%, respectively, *p* < 0.05). Both parameters were restored to the values observed in healthy animals by both types of enzyme supplementation (MD amylase or Creon^®^, *p* < 0.05) (Table 1).

### 2.2. Changes in Brush Border Thickness and Goblet Cell Population in Small Intestine

Thickness of the brush border and goblet cell number and area (normalized per crypt) are shown in Table 2.

The brush border thickness in EPI animals tended to be smaller in the duodenum and proximal and distal jejunum of EPI pigs compared to that of healthy pigs. At the same time, both types of enzyme supplementation (MD amylase or Creon^®^), significantly increased the thickness of the brush border (by 81%, *p* < 0.05).

In the proximal jejunum, MD amylase treatment resulted in the highest values of brush border thickness, which were significantly (*p* < 0.05) higher than those observed in healthy or Creon^®^-treated pigs (Table 2).

In the middle jejunum, the thickness of the brush border was not different between healthy and EPI animals, but was significantly (by 50%, *p* < 0.05) increased by both types of enzyme supplementation (MD amylase or Creon^®^).

In the ileum, a significant (by 47%, *p* > 0.05) decrease in brush border thickness was observed in EPI animals compared to the healthy group, which was completely restored by both MD amylase and Creon^®^ treatment (Table 2).

The number of goblet cells per crypt in the duodenum was not different between EPI and healthy pigs but was significantly (*p* < 0.05) increased by both types of enzyme treatment. MD amylase treatment resulted in the highest numbers of goblet cells per crypt (by 79%, Table 2). A similar situation with regards to the number of goblet cells was observed in the ileal segment (Table 2). Goblet cell number per crypt was unchanged in EPI pigs, with or without Creon^®^ treatment, when compared to the healthy pigs, while MD amylase supplementation resulted in significantly (*p* < 0.05) increased goblet cell numbers in all jejunal segments (proximal, middle, and distal) (by 62%, 89%, and 69%, respectively, Table 2).

At the same time, the area of goblet cells in the duodenum was significantly (by 54%, *p* < 0.05) decreased by EPI compared to healthy animals and the values were restored to those observed in intact pigs by MD amylase, but not Creon^®^ supplementation (Table 2). In the other intestinal segments, no differences were noted between EPI and healthy pigs, but MD amylase significantly increased the area of goblet cells (*p* < 0.05) (Table 2).

Crypt area was significantly (*p* < 0.05) reduced in EPI animals when compared to healthy pigs. In the duodenum, this decrease was partially restored by MD amylase, but not Creon^®^ supplementation, while in the rest of the intestinal segments, no significant effect of any enzyme treatment on the crypt area was observed (Table 2).

### 2.3. The Levels of Accumulated Glycogen in Small Intestinal Mucosa

**Figure 1:**
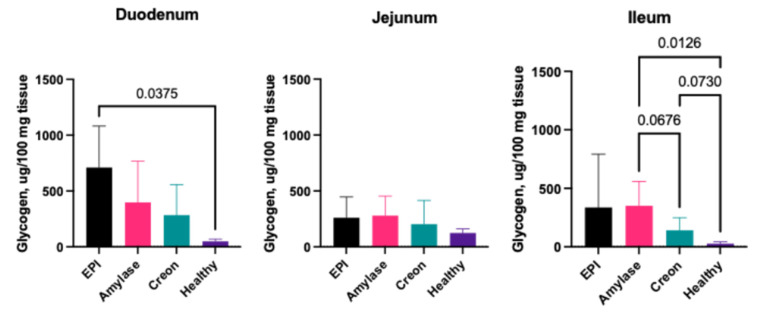
Glycogen accumulation in small intestine. Group EPI Control (n = 6): pigs after PDL fed exclusively an HFD. Group Amylase (n = 6) and Group Creon (n = 6): pigs after PDL fed an HFD + MD amylase (2 × 4000 units per day) or Creon^®^ (2 × 100,000 units per day), respectively, at the morning and evening meals throughout the experiment. Group Healthy (n = 4): intact, healthy pigs fed an HFD. Data are expressed as mean ± standard deviation (±SD). Differences were considered significant at *p* < 0.05; *p*-values are given with the result bars.

Data on glycogen accumulation in small intestinal segments are presented in Figure 1. The levels of glycogen in the duodenal mucosa of healthy animals reached 48.8 ± 21.1 ug/mg tissue, while EPI significantly increased the glycogen accumulation in the duodenum and the levels of this polysaccharide reached 710.6 ± 317.4 ug/mg tissue. No significant effect of either MD amylase or Creon^®^ supplementation was shown (Figure 1).

The glycogen level in the jejunal mucosa of healthy pigs was 124.5 ± 36.1 ug/mg tissue and was not affected by EPI and/or enzyme supplementation (Figure 1). The ileal mucosa glycogen content of healthy, intact pigs was 26.8 ± 16.6 and was significantly increased in the MD amylase-treated group (Figure 1).

### 2.4. The Disaccharidase Activity in Small Intestinal Brush Border

**Figure 2:**
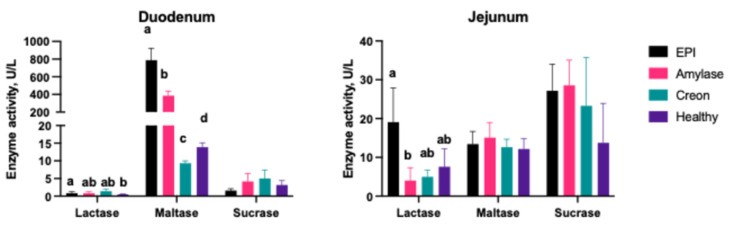
Brush border disaccharidase activity. Group EPI Control (n = 6): pigs after PDL fed exclusively an HFD. Group Amylase (n = 6) and Group Creon (n = 6): pigs after PDL fed an HFD + MD amylase (2 × 4000 units per day) or Creon^®^ (2 × 100,000 units per day), respectively, at the morning and evening meals throughout the experiment. Group Healthy (n = 4): intact, healthy pigs fed an HFD. Data are expressed as mean ± standard deviation (±SD). Different superscript letters given with the result bars describe significance at *p* < 0.05.

Activity of brush border disaccharidases was measured in the duodenal and jejunal segments of the small intestine and the obtained results are shown in Figure 2. The activity of lactase in the duodenal mucosa of healthy pigs was 0.52 ± 0.09 U/L. The pancreatic duct ligation led to a significant (*p* < 0.05) increase in lactase activity, reaching 0.87 ± 0.4 U/L in EPI pigs. No significant effect of either MD amylase or Creon^®^ supplementation on lactase activity in the duodenal mucosa was observed (Figure 2). Brush border maltase activity in the duodenum of healthy pigs was 13.9 ± 1.24 U/L, and a dramatic, 56-fold rise (*p* < 0.05) in maltase activity was observed in EPI pigs. MD amylase supplementation also resulted in increased maltase activity, while Creon^®^ administration led to a decrease in maltase activity when compared to healthy animals (Figure 2). No significant differences in brush border sucrase activity in the duodenum were observed between groups (Figure 2).

In the jejunal segment, no significant differences in the activity of any disaccharidases were observed between healthy and EPI pigs; only a significant (*p* < 0.05) decrease in lactase activity in EPI pigs after MD amylase supplementation was shown (Figure 2).

### 2.5. The Proliferation and Apoptotic Indices in Small Intestinal Segments

**Figure 3:**
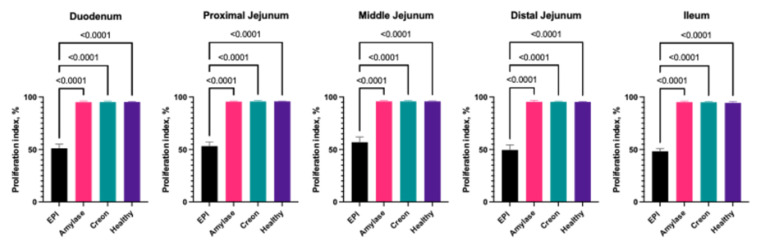
Proliferation index in small intestine. Group EPI Control (n = 6): pigs after PDL fed exclusively an HFD. Group Amylase (n = 6) and Group Creon (n = 6): pigs after PDL fed an HFD + MD amylase (2 × 4000 units per day) or Creon^®^ (2 × 100,000 units per day), respectively, at the morning and evening meals throughout the experiment. Group Healthy (n = 4): intact, healthy pigs fed an HFD. Data are expressed as mean ± standard deviation (±SD). Differences were considered significant at *p* < 0.05; *p*-values are given with the result bars.

**Figure 4:**
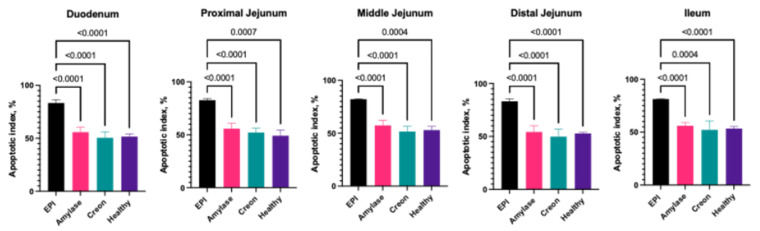
Apoptotic index in small intestine. Group EPI Control (n = 6): pigs after PDL fed exclusively an HFD. Group Amylase (n = 6) and Group Creon (n = 6): pigs after PDL fed an HFD + MD amylase (2 × 4000 units per day) or Creon^®^ (2 × 100,000 units per day), respectively, at the morning and evening meals throughout the experiment. Group Healthy (n = 4): intact, healthy pigs fed an HFD. Data are expressed as mean ± standard deviation (±SD). Differences were considered significant at *p* < 0.05; *p*-values are given with the result bars.

The results of the calculations of the proliferation and apoptotic indices are provided in Figure 3 and Figure 4, respectively. The proliferative (Figure 3) and apoptotic (Figure 4) indices were very similar between all investigated intestinal segments. The proliferation index values in EPI pigs ranged from 48% to 56%, while healthy animals demonstrated values between 94% and 96%. Both types of enzyme supplementation (MD amylase and Creon^®^) completely restored the proliferation of the mucosa cells in the small intestine (Figure 3).

The apoptotic index values in EPI pigs ranged from 81% to 84%, while healthy animals demonstrated values between 49% and 53%. Both types of enzyme supplementation (MD amylase and Creon^®^) completely restored the apoptotic index within the small intestinal segments (Figure 4).

### 2.6. Expression of PepT1

**Figure 5:**
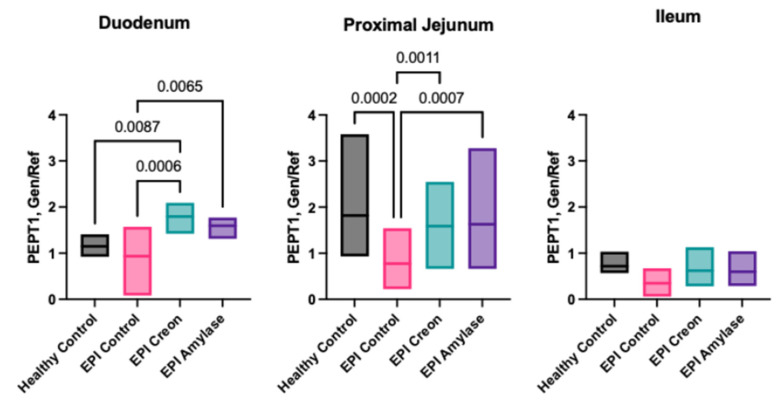
Effect of MD amylase and Creon^®^ supplementation on frequency of *PepT1* transporter in different parts of the gut: duodenum, jejunum, and ileum. Data are expressed as median ± interquartile range (±IQR). The differences between the results were considered significant when *p* < 0.05. *p*-values ranging between 0.1 and 0.05 were considered as a trend. *p*-values are given with the result bars.

Figure 5 shows the peptide transporter 1 (*PepT1*) levels in the gut mucosa of the pigs. MD amylase treatment, as well as Creon^®^, enhanced the expression of *PepT1* throughout the small intestine, compared to that observed in Control EPI pigs. *PepT1* transporter expression in all studied segments of the intestine of EPI pigs reached values similar to those observed in healthy pigs following MD amylase and Creon^®^ treatment.

## 3. Discussion

Porcine pancreatic enzyme supplements are the standard of care for patients with cystic fibrosis (CF) and EPI nowadays. In physician society, dealing with CF patients and obtaining good results with improvement of fat absorption—especially DHA (docosahexaenoic acid) and EPA (eicosapentaenoic acid) [24]—the importance and quantities of proteases and amylase in commonly used pancrelipase formulations are often neglected. It should be mentioned that currently used pancrelipases do not normalize fat absorption in up to 30% of patients and ongoing abdominal complaints are common [25,26]. There have also been concerns about the possible link between porcine pancreatic enzyme preparations and hepatitis E development in CF patients, and shortages have been seen [8,27]. Thus, non-animal sources of digestive enzymes that can be manufactured with modern purity and control standards have been developed. However, to date, no preparation containing exclusively microbial-derived enzymes is available. The aim of the current study was to elucidate the impact of standard-of-care porcine pancreatic enzymes in comparison to microbial-derived alpha amylase on the intestinal wall structure, mucosal glycogen accumulation, and enterocyte turnover in the small intestine.

The impact of exocrine pancreatic insufficiency on intestinal development, microbiota, and function was highlighted in several articles some decades ago [28,29,30,31]. However, most of the above-mentioned studies were conducted on the grounds of PERT, with just a few attempts to look at the changes in the intestinal structure and function provoked by EPI itself, without additional enzyme supplementation [29,30]. Studies that explore biochemical changes and brush border enzyme activities have been shown to be highly species-specific and thus one should consider the relevance of the animal model used. Most previous studies were conducted using dogs with either spontaneously developed or surgically induced EPI or rodent EPI models, and the relevance of these models for translation to human physiology is doubtful.

In the current study, we made use of the well-known and established porcine model of EPI [9,24,32], which possesses the main features of human primary and secondary EPI and has been shown to be a useful tool for the investigation of EPI and possible effects of its enzymatic treatment [24,32,33]. Our findings with regards to the changes in villus length and crypt depth in EPI animals are in line with previous reports by Prykhodko et al. [9]. In the present study, the separation of the intestinal segments was performed more thoroughly than was performed previously (into duodenum, proximal, middle, and distal jejunum and ileum vs. proximal, middle, and distal small intestine), and atrophy varied dependent on segment. Both orally administered MD amylase and a mixture of porcine pancreatic enzymes were able to recover the structure of intestinal segments. The fact that isolated MD amylase was as efficacious as the mixture of lipase, protease, and amylase that is pancrelipase indicates that amylase may have a central role in the preservation of intestinal morphology. Other important parameters, such as mucosa and muscularis thickness, were also estimated and the undoubtful impact of exocrine pancreatic insufficiency on the atrophy of both layers, as well as the possibility of atrophy correction by enzyme supplementation, was demonstrated (Table 1). Previous studies in various EPI animal models have shown the increased permeability of the small intestine, which has even been described for patients with primary EPI due to cystic fibrosis [34,35]. We did not assess the permeability of the intestinal segments in the current study, but could assume that the enzyme-related improvements in intestinal wall structure could possibly benefit the maintenance of its physiological permeability.

We investigated the thickness of the brush border and population of goblet cells as well as glycoprotein accumulation in the intestinal mucosa. The brush border thickness in EPI animals was decreased in the duodenum and jejunum of EPI pigs compared to that of healthy pigs, and this deficit was increased by either MD amylase or Creon (Table 2). Of note, MD amylase treatment resulted in the highest values of brush border thickness in the proximal jejunum. Taken together, these findings suggest that pancreatic enzyme supplementation is important for intestinal integrity in addition to nutrient digestion. Goblet cells and the mucus they secrete serve as an important barrier, preventing pathogens from invading the mucosa to cause intestinal inflammation. Dysbiosis and intestinal inflammation are seen in patients with CF and EPI. Goblet cells are renewed continuously from stem cells at the crypt base. Previous reports from the EPI pig model and humans have shown a significant increase in the goblet cell population in the crypts [9,34]. The authors suggested that such a reaction could be the result of tissue adaptation to bacterial overgrowth and constant intestinal inflammation caused by EPI [31,36,37]. However, we did not observe any significant differences in the number of goblet cells within the crypts or their area between healthy and EPI animals in the current study. Such a discrepancy in the results could be explained by the staining used, as in both mentioned studies, histological techniques, non-specific for glycoproteins, were used, thus making the distinction between goblet cells and enterocytes in the crypts non-reliable. However, we observed a significant increase in goblet cell number and area after amylase treatment. It is worth mentioning that amylase has recently been shown to actively participate in enterocyte turnover [23]; thus, it could probably affect cell differentiation and, in doing so, regulate the number of goblet cells.

An interesting finding was made with regards to the accumulation of glycogen in the intestinal mucosa. It appeared to be minimal in all intestinal segments from healthy pigs and was markedly increased in EPI animals. This is an intriguing observation which could possibly be explained by the adaptation of enterocytes to the lack of digestive enzymes in the intestinal lumen, and, thus, energy deficit, which could not be restored quickly during digestion. In such a way, this possible deficit of energy could be successfully prevented by the storage of glycogen. Previously, it was reported that enterocytes can accumulate glycogen as a result of altered glucose metabolism and absorption pathways when energy availability is compromised [38]. A study by Karasov et al. [39] highlighted that pancreatic enzyme deficiencies lead to a cascade of metabolic adjustments in the gastrointestinal tract. These include upregulation of glycogen synthase activity, increased gluconeogenesis, and other compensatory mechanisms to maintain homeostasis in the face of enzyme-related malabsorption. This is supported by our own findings regarding brush border disaccharidase activity presented in this study. The pattern of maltase activity changes in the duodenum of healthy and EPI pigs, with and without enzyme supplementation, reflects the pattern of glycogen accumulation in the small intestine—especially in the duodenum (Figure 1 and Figure 2). Therefore, one could reason that during periods of general malnutrition and lack of building materials (protein), the enterocytes attempt to store glucose in the form of glucagon for a “better” metabolic time, while at the same time protecting the whole body from hyperglycemia.

Enterocyte turnover was also very different from previously described findings in a rat model [29], where increased enterocyte turnover was shown. We demonstrated that EPI in pigs results in significantly increased apoptotic activity, while proliferative activity is dramatically decreased in comparison to healthy animals. Apoptosis is a tightly regulated process that removes damaged or unneeded cells. In the context of EPI, the lack of pancreatic enzymes leads to malabsorption of nutrients, which can cause cellular stress and damage within the intestinal mucosa. This stress may activate pro-apoptotic pathways, increasing the rate of cell death. In EPI, the energy deficit caused by malabsorption might impair the cell’s ability to repair itself, leading to increased rates of apoptosis. Moreover, the absence of pancreatic enzymes likely results in the incomplete digestion of dietary components, contributing to dysbiosis, which could further damage the intestinal lining, exacerbating cell death. This heightened apoptotic activity might contribute to mucosal atrophy and decreased intestinal function, as cells are not being replaced at a sufficient rate due to diminished proliferative activity. Surprisingly, both the mixture of porcine-derived pancreatic enzymes and the microbial amylase supplementation resulted in the complete recovery of proliferative and apoptotic activity in all investigated intestinal segments (Figure 3 and Figure 4). Several studies have supported the role of pancreatic enzyme supplementation in improving intestinal health in EPI. A study on dogs with EPI found that enzyme replacement significantly reduced mucosal atrophy and improved villus height, which was correlated with reduced apoptosis and increased proliferative activity [40]. Lammers et al. [41] investigated the impact of pancreatic enzyme supplementation on growth factors in the gut and found that PERT enhances the production of EGF and TGF-β, both of which are key regulators of intestinal cell proliferation and apoptosis. A study by de Oliveira [42] demonstrated that microbial amylase supplementation in EPI patients improved carbohydrate digestion and absorption, which was associated with reduced gut inflammation and improved epithelial integrity. Along with our data, this supports the idea that even partial restoration of enzyme function can significantly improve intestinal health by modulating apoptosis and proliferation.

Our data (Figure 5) point out that both MD amylase and Creon^®^ treatment similarly stimulate *PepT1* expression in the intestinal mucosa of young, EPI pigs. It is possible that the reported improvement in peptide absorption in pigs treated with MD amylase could be explained by the lack of utilization of peptides by the gut mucosa and their enhanced absorption because of the increased expression of *PepT1* transporters observed following MD amylase treatment. At the same time, the action of Creon^®^ proteases results in the release of free amino acids, which are the preferable source of fuel for enterocytes [43]. Interestingly, the question remains as to whether peptide absorption is accomplished exclusively via *PepT1*. Recent findings by Rohm et al. [44] have clearly shown that the number of plasma peptides present after a meal cannot be attributed only to the transport ability of *PepT1* [44]. Therefore, it is even possible that pancreatic enzymes participate in the regulation of other peptide transport mechanisms such as the paracellular pathway. Mechanisms of enhanced protein absorption independent of *PepT1* remain to be explored.

## 4. Materials and Methods

### 4.1. Animal Experiment and Diets

#### 4.1.1. Animals

The study received approval from the Second Local Ethical Committee for Animal Experiments in Warsaw (approval number WAW2/025/2022). All procedures involving animals were performed in accordance with the Polish Law for the Care and Use of Animals, EU regulations (Directive 2010/63/EU) and the Code of Ethics of the World Medical Association (Declaration of Helsinki). The current experiment complied with the ARRIVE guidelines. Sample size was estimated using G∗Power software, version 3.1.9.4 [45] for a one-way ANOVA at α = 0.2 with 80% power, assuming f (effect size) = 0.95, for four study groups. The experiment was conducted on 22 hybrid pigs ((Polish Spruce × Yorkshire) × Hampshire breed) of both sexes, whose body weight at the beginning of the study was 15.0 ± 2.3 kg, and then the animals were weighed once a week. The animals were kept individually in floor boxes in which they had constant visual, olfactory, and tactile contact with each other. The animals had constant access to fresh water throughout the experiment. Zootechnical conditions were standard for an experimental pig facility (light cycle 12/12, temperature 21–25 °C, humidity 70 +/− 5%, air exchanges 10–12/h).

#### 4.1.2. Feeding and Enzyme Administration

**Figure 6:**
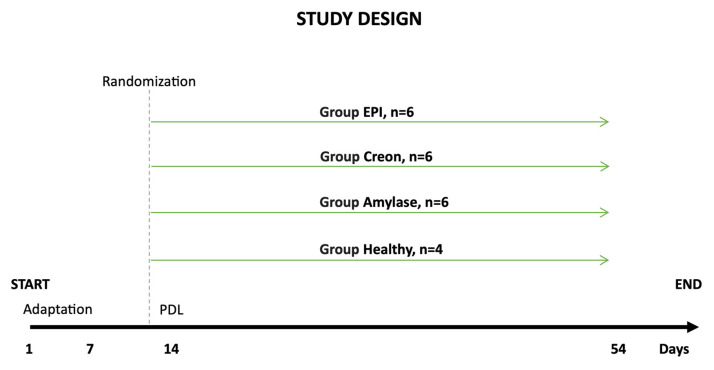
Study design. Days 1–7—adaptation period after arrival to animal facility; days 8–13—adaptation of pigs to a high-fat diet (HFD). On day 13, pigs were randomized and kept on respective diets throughout the experimental period. Pancreatic duct ligation (PDL) surgery was performed on day 14. After complete EPI development on day 54, the experiment ended.

Upon arrival at the experimental unit housing, the pigs were fed a cereal-based, pelleted, standard diet, which was gradually changed by day 5 to a high-fat diet (HFD) containing 17.5% crude protein, 3.9% crude fiber, 20% crude fat, and 5.2% ash, together with 5000 IU/kg vitamin A, 500 IU/kg vitamin D, and 85 mg/kg vitamin E. The HFD (Kcynia, Morawski Plant, Lodz, Poland) was equivalent to 4% of their body weight per day, of which 1% was fed in the morning meal (08:00–09:00) and 3% in the afternoon meal (16:00–17:00). MD amylase DS100 (Amano Enzymes, *Aspergillus* oryzae, 4000 units/dose) or Creon^®^ 25000 (Abbot, IL, USA, 100,000 units/dose) was administered to pigs with morning and evening meals, depending on the randomization. Randomization was performed on day 13 of the experiment (based on average body weight), after the end of the adaptation period (day 7) and before pancreatic duct ligation (PDL) surgery (day 14). Group EPI (n = 6): pigs after PDL fed exclusively an HFD. Group Amylase (n = 6) and Group Creon (n = 6): pigs after PDL fed an HFD + MD amylase (2 × 4000 units per day) or Creon^®^ (2 × 100,000 units per day), respectively, at the morning and evening meals throughout the experiment. Group Healthy (n = 4): intact, healthy pigs fed an HFD. The detailed study design is shown in Figure 6.

#### 4.1.3. Autopsy

At the end of the study, all pigs were euthanized by a single dose of intravenously injected sodium pentobarbiturate (140 mg/kg bwt) (“Morbital”, Biowet, Pulawy, Poland), after prior intravenous premedication with a mixture of medetomidine (7 mcg/kg bwt) (“Domitor”, Orion Pharma, Warsaw, Poland) and butorphanol (0.2 mg/kg bwt) (“Torbugesic”, Zoetis, Warsaw, Poland).

### 4.2. Histomorphometric Analysis

On autopsy, samples from the duodenum, proximal, middle, and distal parts of the jejunum and ileum were collected and fixed in 10% neutral buffered formalin for 24 h. Before deposition, cross-sections of tissue samples (approximately 5 mm) containing all structural layers were cut from a 3-centimeter-long sample. Then, the tissue fragments were dehydrated and embedded in paraffin according to standard histological techniques [46]. Paraffin-embedded tissues were cut into 4.5-micrometer-thick sections using a rotor microtome. After drying overnight, the slides were deparaffinized by incubation in xylene and decreasing concentrations of ethanol. Sections were used either for standard hematoxylin and eosin staining [47] to estimate parameters such as the thickness of the muscular lamina and mucosa, the length of the villi, and the depth of the crypts, or Periodic Acid–Schiff (PAS) Staining to assess the brush border thickness and goblet cell population [48]. The analysis was performed using a light microscope (Axioskop 40, Zeiss, Jena, Germany) equipped with a digital camera (Coolpix B700, Nikon, Tokyo, Japan). The obtained data were accessed using Axio Vision software version 4.2 (Zeiss, Germany). A minimum of 20 measurements of each parameter were made for each section.

### 4.3. Immunohistochemical Analysis

#### 4.3.1. Intestinal Crypt Stem Cell Proliferative Activity

For immunohistochemical analysis, sections were dehydrated in an alcohol series and washed with 0.1 M PBS (PBS tablets, Medicago, Sweden). The sections were then placed in boiling citrate buffer (10 mM citric acid, 0.05% Tween 20, pH 6.0) for 10 min, washed with 0.1 M PBS, and incubated for 5 min in 0.3% H_2_O_2_ solution. Sections were then rinsed with 0.1 M PBS and primary antibodies against PCNA (proliferating cell nuclear antigen) were applied (mouse monoclonal anti-PCNA [pc10] Abcam, Cambridge, UK) at a dilution of 1:1000. After 30 min of incubation in a humid chamber at room temperature, sections were rinsed twice with 0.1 M PBS and incubated in PBS for 5 min during the final rinse. A mouse-specific HRP polymer (Envision+, Dako, Glostrup, Denmark) was used to detect primary antibodies. Sections were incubated for 30 min in a humid chamber at room temperature and then washed twice with 0.1 M PBS. After this step, 3,30-diaminobenzidine substrate (DAB, Envision+, Dako, Glostrup, Denmark) was applied and the sections were incubated for 15 min, then rinsed with distilled water and stained with hematoxylin to visualize the nuclei. Sections were mounted with Permount mounting medium (S3025, Dako, Glostrup, Denmark) and the mitotic index was calculated as the percentage of PCNA-positive cells (3,30-diaminobenzidine staining, brown) among all epithelial cells in the crypt section. For each section, a minimum of 20 measurements were made under a light microscope (Axioskop 40, Zeiss, Germany, magnification—400×).

#### 4.3.2. Apoptotic Index of the Epithelial Cells of the Small Intestine

The ApopTag^®^ Peroxidase In Situ Apoptosis Detection Kit (S7100, Chemicon, Rolling Meadows, IL, USA) was used to detect and label apoptotic cells in the small intestinal epithelium. Sections were placed in boiling citrate buffer (10 mM citric acid, 0.05% Tween 20, pH 6.0) for 10 min and then washed with 0.1 M PBS. The procedure recommended by the manufacturer was then followed. Sections were mounted using a Paramount (S3025, Dako, Glostrup, Denmark). The apoptotic index was assessed as the percentage of apoptotic cells among the total number of villus epithelial cells (1/3 of the villus length from the villus apex). A minimum of 20 measurements were made for each section, and tissue images were analyzed using a light microscope (Axioskop 40, Zeiss, Germany, magnification—100×).

### 4.4. Glycogen Determination

On autopsy, mucosa samples from the duodenum, proximal, middle, and distal parts of the jejunum and ileum were collected and immediately frozen at −80 °C for further analysis of glycogen content. Glycogen was extracted from intestinal mucosa by alkaline hydrolysis using the Good–Kramer–Somogyi modification of Pfluger’s method [49]. Glycogen content in the obtained extracts was measured using a glycogen assay kit MAK016 (Sigma-Aldrich, Saint Louis, MO, USA), according to the manufacturer’s instructions.

### 4.5. Brush Border Disaccharidase Activity Assay

Small intestinal mucosa samples were homogenized in ice-cold, 0.9% NaCl (1:25, *w/v*) using a glass homogenizer. The disaccharidase, lactase, maltase, and sucrase activities were measured in accordance with the Dahlqvist assay [50]. In parallel, the total protein content in the intestinal homogenates was determined by the Lowry method [51], using purified BSA as the standard. The disaccharidase activities were recalculated per mg of total protein. All reagents were purchased from Sigma-Aldrich, Saint Louis, MO, USA.

### 4.6. Expression of PepT1

To determine the effects of the pancreatic enzymes on the relative mRNA expression level of the *PepT1* gene along the gastrointestinal tract, total RNA was isolated from the intestinal mucosa and real-time PCR was performed according to the previous protocol [52]. Total mRNA from collected tissues was isolated using the Total RNA Mini Kit (A&A Biotechnology, Gdynia, Poland) according to the manufacturer’s protocol. The yield of isolated RNA was assessed spectrophotometrically (Nanodrop, NanoDrop Technologies, Wilmington, DE, USA) and integrity was assessed electrophoretically by separation on a 1.5% agarose gel containing ethidium bromide. To synthesize complementary cDNA, 1000 ng/mL mRNA from selected tissues in a total volume of 20 μL was retrotranscribed using the Maxima First Strand cDNA Synthesis Kit for RT-qPCR, with ds DNase (ThermoFisher Scientific, Warsaw, Poland) according to the manufacturer’s instructions. The pig (Sus scrofa domestica)-specific primers used for housekeeping and test gene expression determination (*PepT1*, *ACTB, GADPH, B2M;* Table 3) were designed using Primer-designing tool NCBI software (National Library of Medicine, Bethesda, MD, USA; https://www.ncbi.nlm.nih.gov/tools/primer-blast/) and synthesized by Genomed (Poland). Real-time qPCR was performed using 5× HOT FIREPol EvaGreen^®^ qPCR Mix Plus (ROX) (Solis BioDyne, Tartu, Estonia) in a total volume of 20 μL containing 4 μL qPCR Mix Plus, 13 μL H_2_O PCR grade, 2 × 0.5 μL primers (0.5 mM), and 2 μL cDNA template. Amplification was performed using a Rotor Gene 6000 thermocycler (Corbett Research, Mortlake, Australia) according to the following PCR protocol: one cycle at 95 °C for 15 min (enzyme activation); forty cycles at 95°C for 15 s (denaturation), 60 °C for 20 s (annealing), and 72 °C for 20 s (elongation); followed by one cycle at 72 °C for 7 min (product stabilization). The melting curve was performed at 70–95°C in 0.5 °C intervals. Negative controls without the cDNA template were included in each reaction. The real-time qPCR reaction for each cDNA sample was performed in duplicate. The identity of the PCR products was confirmed by direct sequencing. Relative gene expression was calculated using the comparative quantification option of Rotor Gene 6000 1.7 software (Qiagen GmbH, Hilden, Germany) and determined using the Relative Expression Software Tool (http://rest.gene-quantification.info/) according to the PCR efficiency correction algorithm. Expression of the analyzed genes was normalized to the expression of chosen housekeeping genes *ACTB*, *GADPH*, and *B2M*. The Bestkeeping method was used to determine the most stable housekeeping gene for normalizing the expression of the genes of interest. The Bestkeeping method is based on pairwise correlation analysis of all pairs of candidate genes. The primer sequences are listed in Table 3.

### 4.7. Statistical Analysis

Statistical analysis was performed on the data generated from this study using the Brown–Forsythe and Welch ANOVA for normally distributed datasets or Kruskal–Wallis test when data were not normally distributed. The data distribution was assessed using the Shapiro–Wilk normality test. Outliers within datasets were identified using the ROUT method of regression, using Q = 0.05%. All the analyses were carried out using GraphPad Prism 10.3.0 (461), San Diego, CA, USA. Data were not corrected for multiple comparisons. Differences were considered significant if *p* ≤ 0.05; differences were considered as a trend when *p* ≤ 0.1; data with a Gaussian distribution are expressed as mean ± standard deviation (±SD), and data with a non-Gaussian distribution are expressed as median ± interquartile range (±IQR).

## 5. Limitations of the Study

The main limitation of our study is the difference between the porcine and human digestive system. Despite many similarities, there are differences in carbohydrate digestion, enzyme profiles, different length and absorption area of small intestine, strong dependency of intestinal metabolism on amino acids in pigs, and differences in intestinal trophic response, among others. Moreover, the mechanisms underlying our findings require thorough further investigation. However, the patterns of mucosal maturation, cell differentiation, and development of tissue architecture from proximal to distal regions of the small intestine are very similar between pigs and human beings [53], thus allowing us to extrapolate our results to human physiology.

## 6. Conclusions

To our knowledge, this is the first manuscript describing changes in small intestinal structure of EPI pigs before and after treatment with exogenous digestive enzymes. Our findings provide support for the importance of enzymes—especially amylase—in the gut for the maintenance of physiological enterocyte turnover and intestinal wall architecture in addition to the digestion of nutrients. We have shown the amelioration of mucosal atrophy by enzyme treatment, thus emphasizing the importance of enzyme treatment for the intestinal health.

## Figures and Tables

**Table 1:** Morphological parameters measured in the small intestinal segments of experimental animals.

Parameter/Group	EPI	Amylase	Creon	Healthy
Duodenum				
Mucosa thickness, μm	1207.0 ± 132.3	1374.0 ± 191.5	1373.0 ± 165.1	1288.0 ± 36.8
Villus length, μm	662.1 ± 113.3 ^a^	863.7 ± 120.1 ^b^	984.7 ± 174.0 ^b^	857.5 ± 41.5 ^b^
Crypt depth, μm	171.4 ± 9.5 ^a^	249.6 ± 42.5 ^b^	247.8 ± 25.0 ^b^	227.2 ± 31.5 ^b^
Muscularis thickness, μm	174.0 ± 36.8 ^a^	304.7 ± 40.7 ^b^	317.4 ± 27.2 ^b^	326.8 ± 48.3 ^b^
Proximal Jejunum				
Mucosa thickness, μm	1086.0 ± 145.1	1300.0 ± 194.4	1145.0 ± 95.8	1153.0 ± 40.4
Villus length, μm	605.9 ± 106.4 ^a^	828.6 ± 143.5 ^b^	744.3 ± 80.6 ^b^	777.8 ± 36.9 ^b^
Crypt depth, μm	174.8 ± 6.2 ^a^	234.3 ± 19.3 ^b^	216.5 ± 24.6 ^b^	225.1 ± 16.8 ^b^
Muscularis thickness, μm	210.5 ± 71.7 ^a^	265.6 ± 32.2 ^ab^	295.0 ± 21.7 ^b^	293.1 ± 45.6 ^b^
Middle Jejunum				
Mucosa thickness, μm	975.9 ± 91.6 ^ac^	1095.0 ± 85.6 ^ab^	1107.0 ± 53.7 ^b^	958.4 ± 48.6 ^c^
Villus length, μm	569.2 ± 75.4 ^a^	681.5 ± 85.6 ^ab^	706.2 ± 32.8 ^b^	648.8 ± 29.6 ^a^
Crypt depth, μm	188.5 ± 19.9 ^a^	215.8 ± 25.0 ^b^	244.2 ± 23.0 ^c^	199.2 ± 18.9 ^ab^
Muscularis thickness, μm	189.5 ± 47.7 ^a^	302.2 ± 31.2 ^b^	313.9 ± 23.6 ^b^	308.9 ± 18.1 ^b^
Distal Jejunum				
Mucosa thickness, μm	839.5 ± 101.2	949.8 ± 99.3	948.4 ± 75.1	904.4 ± 47.1
Villus length, μm	501.8 ± 82.5 ^a^	609.9 ± 106.2 ^ab^	666.2 ± 81.6 ^b^	586.3 ± 133.1 ^a^
Crypt depth, μm	181.0 ± 27.6 ^a^	230.9 ± 43.2 ^b^	233.6 ± 20.1 ^b^	222.3 ± 63.3 ^a^
Muscularis thickness, μm	189.7 ± 54.0 ^a^	306.3 ± 45.0 ^b^	327.9 ± 25.5 ^b^	295.2 ± 93.0 ^ab^
Ileum				
Mucosa thickness, μm	764.8 ± 140.6	841.6 ± 94.6	878.7 ± 137.3	844.5 ± 24.8
Villus length, μm	470.1 ± 109.4	539.6 ± 91.6	544.0 ± 101.1	591.5 ± 50.9
Crypt depth, μm	164.7 ± 7.2 ^a^	204.2 ± 34.6 ^b^	213.3 ± 22.0 ^b^	214.6 ± 9.0 ^b^
Muscularis thickness, μm	198.4 ± 62.8 ^a^	326.3 ± 93.4 ^b^	325.9 ± 64.8 ^b^	286.1 ± 32.2 ^b^

Group EPI Control (n = 6): pigs after PDL fed exclusively an HFD. Group Amylase (n = 6) and Group Creon (n = 6): pigs after PDL fed an HFD + MD amylase (2 × 4000 units per day) or Creon^®^ (2 × 100,000 units per day), respectively, at the morning and evening meals throughout the experiment. Group Healthy (n = 4): intact, healthy pigs fed an HFD. Data are expressed as mean ± standard deviation (±SD). Different superscript letters given with values within a row describe significance at *p* < 0.05. Tendencies for differences when *p* < 0.1 are only mentioned in the text.

**Table 2:** Brush border thickness and goblet cell population characteristics in the small intestinal segments of experimental animals.

Parameter/Group	EPI	Amylase	Creon	Healthy
Duodenum				
Brush border thickness, μm	4.3 ± 1.5 ^a^	7.8 ± 2.0 ^b^	7.8 ± 1.0 ^b^	5.7 ± 1.5 ^ab^
Goblet cell area in crypts, μm^2^	1602.0 ± 426.0 ^a^	4419.0 ± 853.5 ^b^	2374.0 ± 776.1 ^a^	3421.0 ± 666.9 ^b^
Crypt area, μm^2^	9892.0 ± 3357.0 ^a^	14,495 ± 2554 ^b^	12,335 ± 2247 ^a^	21,424 ± 3447 ^c^
Goblet cells, n per crypt	8.2 ± 0.8 ^a^	14.7 ± 1.1 ^b^	10.9 ± 1.0 ^c^	10.4 ± 1.9 ^ac^
Proximal Jejunum				
Brush border thickness, μm	4.4 ± 1.9 ^a^	8.3 ± 2.3 ^b^	7.1 ± 1.4 ^c^	5.7 ± 1.2 ^ac^
Goblet cell area in crypts, μm^2^	1809.0 ± 692.2 ^a^	3577.0 ± 705.1 ^b^	2184.0 ± 309.8 ^a^	2511.0 ± 601.6 ^a^
Crypt area, μm^2^	11,318 ± 4287 ^a^	13,615 ± 2510 ^a^	14,142 ± 2805 ^a^	20,174 ± 2739 ^b^
Goblet cells, n per crypt	8.3 ± 0.6 ^a^	13.9 ± 1.6 ^b^	9.7 ± 0.8 ^a^	8.6 ± 1.5 ^a^
Middle Jejunum				
Brush border thickness, μm	5.6 ± 1.5 ^a^	8.4 ± 1.0 ^b^	8.6 ± 1.0 ^b^	6.4 ± 0.6 ^a^
Goblet cell area in crypts, μm^2^	1989.0 ± 801.8 ^a^	3152.0 ± 677.6 ^b^	2331.0 ± 572.4 ^a^	3122.0 ± 670.0 ^ab^
Crypt area, μm^2^	14,333 ± 5451 ^a^	12,728 ± 2841 ^a^	16,053 ± 2908 ^a^	22,584 ± 3935 ^b^
Goblet cells, n per crypt	7.9 ± 1.7 ^a^	14.9 ± 1.5 ^b^	10.2 ± 1.9 ^a^	8.3 ± 1.6 ^a^
Distal Jejunum				
Brush border thickness, μm	4.1 ± 1.4 ^a^	7.2 ± 2.3 ^b^	7.4 ± 1.0 ^b^	5.8 ± 1.2 ^ab^
Goblet cell area in crypts, μm^2^	1736.0 ± 778.7 ^a^	3622.0 ± 798.8 ^b^	2556.0 ± 459.0 ^a^	2476.0 ± 425.5 ^a^
Crypt area, μm^2^	10,713 ± 4930 ^a^	12,381 ± 4573 ^ab^	13,425 ± 1250 ^ab^	17,155 ± 2747 ^b^
Goblet cells, n per crypt	8.9 ± 1.0 ^a^	15.0 ± 1.6 ^b^	9.9 ± 1.1 ^a^	8.8 ± 0.9 ^a^
Ileum				
Brush border thickness, μm	3.4 ± 1.6 ^a^	8.4 ± 1.4 ^b^	6.8 ± 1.6 ^b^	6.4 ± 1.4 ^b^
Goblet cell area in crypts, μm^2^	2335.0 ± 1363.0 ^a^	4598.0 ± 728.8 ^b^	2626.0 ± 230.0 ^a^	3659.0 ± 820.8 ^ab^
Crypt area, μm^2^	11,744 ± 6474 ^a^	13,955 ± 2809 ^a^	13,360 ± 912 ^a^	22,407 ± 5307 ^b^
Goblet cells, n per crypt	10.1 ± 1.6 ^a^	16.5 ± 3.5 ^b^	12.1 ± 1.6 ^c^	10.3 ± 0.3 ^a^

Group EPI Control (n = 6): pigs after PDL fed exclusively an HFD. Group Amylase (n = 6) and Group Creon (n = 6): pigs after PDL fed an HFD + MD amylase (2 × 4000 units per day) or Creon^®^ (2 × 100,000 units per day), respectively, at the morning and evening meals throughout the experiment. Group Healthy (n = 4): intact, healthy pigs fed an HFD. Data are expressed as mean ± standard deviation (±SD). Different superscript letters given with values within a row describe significance at *p* < 0.05. Tendencies for differences when *p* < 0.1 are only mentioned in the text.

**Table 3:** Genes and primers used in the study.

Gene	Acc. No	Primer Sequence (5′–3′)	Amplicon Size
*PepT1*	EU400159	CATCGCCATACCCTTCTG TTCCCATCCATCGTGACATT	144
*GAPDH*	X94251	ACACTCACTCTTCTACCTTTG CAAATTCATTGTCGTACCAG	90
*ACTB*	AY550069	TGCGGCATCCACGAAACTAC AGGGCCGTGATCTCCTTCTG	146
*B2M*	NM_213978.1	ACTTTTCACACCGCTCCAGT CGGATGGAACCCAGATACAT	180

Abbreviations: *PepT1*—Peptide transporter 1; *GADPH*—Glyceraldehyde 3-phosphate dehydrogenase; *ACTB*—β-actin; *B2M*—β2 microglobulin.

## Data Availability

All data generated during the study are available from the corresponding author upon reasonable request.
